# The Sweet Side of HIPK2

**DOI:** 10.3390/cancers15102678

**Published:** 2023-05-09

**Authors:** Alessia Garufi, Valerio D’Orazi, Giuseppa Pistritto, Mara Cirone, Gabriella D’Orazi

**Affiliations:** 1 Unit of Cellular Networks, Department of Research and Advanced Technologies, IRCCS Regina Elena National Cancer Institute, 00144 Rome, Italy; alessiagarufi@yahoo.it; 2 Department of Surgery, Sapienza University, 00185 Rome, Italy; valerio.dorazi@uniroma1.it; 3 Centralized Procedures Office, Italian Medicines Agency (AIFA), 00187 Rome, Italy; pistritto@med.uniroma2.it; 4 Laboratory Affiliated to Pasteur Institute Italy Foundation Cenci Bolognetti, Department of Experimental Medicine, Sapienza University of Rome, 00161 Rome, Italy; mara.cirone@uniroma1.it; 5 Department of Neurosciences, Imaging and Clinical Sciences, University “G. D’Annunzio”, 66013 Chieti, Italy

**Keywords:** hyperglycemia, diabetes, p53, apoptosis, drug resistance, HIF-1, hypoxia, microRNA, cancer, diabetic complications

## Abstract

**Simple Summary:**

Hyperglycemia is a hallmark of diabetes mellitus and contributes to several complications of this disease, such as diabetic retinopathy, diabetic nephropathy, and diabetic foot ulcer. Epidemiological evidence showed that patients suffering from diabetes are at a significantly higher risk for the development of cancer; in addition, the high glucose condition can reduce the efficacy of anticancer therapies. Therefore, uncovering novel molecular mechanisms deregulated by high glucose could give insights into the progression of high glucose-related pathologies other than the development of more efficient anticancer therapies. In this regard, homeodomain-interacting protein kinase 2 (HIPK2) has recently been disclosed in both high glucose-induced cancer resistance to chemotherapies and in diabetic complications, which will be summarized in the present review.

**Abstract:**

HIPK2 is an evolutionary conserved protein kinase which modulates many molecular pathways involved in cellular functions such as apoptosis, DNA damage response, protein stability, and protein transcription. HIPK2 plays a key role in the cancer cell response to cytotoxic drugs as its deregulation impairs drug-induced cancer cell death. HIPK2 has also been involved in regulating fibrosis, angiogenesis, and neurological diseases. Recently, hyperglycemia was found to positively and/or negatively regulate HIPK2 activity, affecting not only cancer cell response to chemotherapy but also the progression of some diabetes complications. The present review will discuss how HIPK2 may be influenced by the high glucose (HG) metabolic condition and the consequences of such regulation in medical conditions.

## 1. Introduction

Hyperglycemia is an excess of glucose concentration in the blood which occurs when the pancreas produces too little insulin or when the body cannot use the insulin properly. The first condition is referred to as type 1 diabetes (T1D), while the second condition is referred to as type 2 diabetes (T2D) [1]. Hyperglycemia is a hallmark of diabetes mellitus and contributes to the development of several complications of this disease, such as diabetic retinopathy (DR), diabetic nephropathy (DN), and impairment of the angiogenesis/wound healing process leading to diabetic foot ulcer [2,3,4]. Medical conditions such as obesity, pancreatitis, chronic stress, and cancer can also be accompanied by hyperglycemia [5,6,7,8]. In addition, hyperglycemia can predispose individuals to cancer development and progression, as evidenced by epidemiologic evidence showing that patients affected by diabetes mellitus are at a higher risk of developing many types of cancers; in addition, the high glucose (HG) condition may promote cancer cell resistance to anticancer chemotherapies [7,8,9]. The link between obesity, T2D, and cancer is quite complex. It involves the production of pro-inflammatory cytokines leading to insulin resistance and to a chronic inflammation status which favor DNA damage and, therefore, cancer development [10]. Hyperglycemia can also reduce dendritic cell (DC) differentiation with impairment of the immune response, reducing the pressure of the immune system against carcinogenesis and infection [11]. The HG condition can induce chemoresistance through the modulation of several molecular pathways. In this regard, a link between HG and homeodomain-interacting protein kinase 2 (HIPK2) has recently been disclosed in both cancer resistance to chemotherapies and in diabetic complications, which will be summarized in the present review.

## 2. HIPK2 Function and Dysfunction

HIPK2 is a highly conserved nuclear serine/threonine kinase member of the corepressors for homeodomain transcription factors—HIPK—family (numbered 1–4) [12], related to the dual-specificity tyrosine phosphorylation regulated kinases (DYRK) [13]. HIPK1, 2, and 3 share 90% of their homology, suggesting, at least in part, functional redundancy [14]; HIPK4 is, until now, the least characterized member of the family [15]. HIPK2 is the best characterized member of the family: its structure comprises an N-terminal region containing a sumoylation site and a kinase domain, a homeobox-interacting domain and a C-terminal region which includes a speckle-retention signal, a putative autoinhibitory domain, and an ubiquitylation site [16]. HIPK2 acts as transcriptional regulator of critical signal transduction pathways, including Wnt/β-catenin, transforming growth factor (TGF)-β, MAPK, and Notch [17], and can negatively or positively regulate gene transcription [18]. Finally, HIPK2 may also have a role in chromatin compaction [19]. As a consequence of the interplay with multiple molecular pathways, HIPK2 regulates basic cellular functions, such as cell proliferation, apoptosis, DNA damage response, cytokinesis, protein stability, and transcription [18,20,21,22,23]. These basic functions of HIPK2 may explain its key role in several biological processes, including development, fibrosis, angiogenesis, and neurodegeneration [24,25,26,27,28], as well as its function as a “bona fide” tumor suppressor [16].

**Figure 1:**
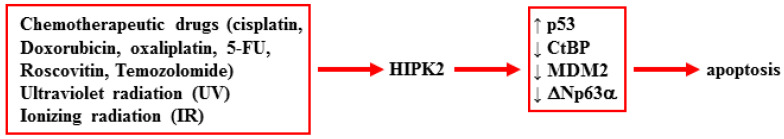
Schematic representation of HIPK2 apoptotic activity. Following activation by drugs and radiations (UV and IR) (red arrow), HIPK2 activates (↑) or inhibits (↓) molecules involved in apoptosis regulation, inducing apoptosis.

In the cancer field, HIPK2 plays a key role in inducing cancer cell apoptosis by modulating several different molecules. HIPK2 is activated in response to chemotherapeutic drugs (including cisplatin, doxorubicin, oxaliplatin, roscovitin, 5-FU, temozolomide) or by ultraviolet (UV) and ionizing radiations (IR). Once activated, HIPK2 phosphorylates the tumor suppressor p53 at serine 46 (Ser46) [20,29], the anti-apoptotic co-repressor CtBP [30], the p53 inhibitor MDM2 [31], or the pro-survival dominant negative isoform of the p53 family member p63 (ΔNp63α) [32], leading to cancer cell apoptosis (Figure 1). Tumor suppressor p53 is the most important target of HIPK2; thus, the inhibition of HIPK2 (e.g., by small interfering (si) RNA) impairs p53 function [33] and cancer cell response to anticancer therapies [34].

HIPK2 expression, stability, subcellular localization (nuclear and/or cytoplasmic), and activity are tightly regulated by post-translational modifications such as polyubiquitination, SUMOylation, phosphorylation, and acetylation [35,36,37]. The HIPK2 post-translational modifications, in response to different types and intensity of stress, may modify its function. For instance, ROS-induced HIPK2 acetylation has been shown to control the survival threshold to oxidative stress; thus, an acetylation-mimicking HIPK2 variant can promote cell survival even under conditions of high oxidative stress [38]. In addition, it has been shown using an in vivo Drosophila model that, in response to a high sugar diet, O-GlcNAc transferase (OGT) induces Hipk O-GlcNAcylation, which prevents Hipk from proteasomal degradation leading to Hipk-induced tissue growth abnormalities [39]. This is in line with a previous study showing that overexpression of Hipk in Drosophila promotes tissue growth abnormalities and several tumor-like features [40]. The authors showed that the regulation of Hipk protein stability by OGT is conserved in human HIPK2, in an in vitro cell system, underscoring how HIPK2 stability and function might be affected by metabolic cues through the nutrient sensor OGT [39]. Intriguingly, it has been shown that the high protein levels of HIPK2 in a mouse model of diabetes [41] depend on the downregulation of E3 ubiquitin ligase Siah-1 (seven in absentia homolog-1), which is known to induce HIPK2 protein degradation [42,43]. Hence, the nutritional regulation of HIPK2 might be achieved by multiple strategies involving direct O-GlcNAcylation and impairment of proteasomal degradation. Cancer cells often sustain high rates of glucose uptake aided by metabolic reprogramming [44]. Thus, an ample supply of glucose not only facilitates aerobic glycolysis within tumors but also induces hyper–O-GlcNAcylation, as seen in several cancers, including breast, prostate, colon, lung, liver, pancreatic, and leukemias [45]. Since elevated levels of HIPK2 have been found to be associated with malignancy of pilocytic astrocytomas [46] and cervical carcinogenesis [47], it is tempting to speculate that the dynamic control of HIPK2 abundance by O-GlcNAc modification can be exploited in the treatment of HIPK2-related disorders with the use of dietary control or metabolic drugs targeting the hexosamine biosynthetic pathway (HBP)–OGT axis. In this regard, it is worth further studying how the HIPK2 post-translational modifications by, for instance, environmental cues such as nutrients may differentially affect its function in normal and in tumoral growth.

Until now, only a few mechanisms have been discovered that lead to HIPK2 inactivation. One mechanism of HIPK2 inactivation is its cytoplasmic localization that occurs, for instance, following integrin alpha(6)beta(4) overexpression [48] or by Src-induced HIPK2 phosphorylation [49]. Altered integrin alpha(6)beta(4) expression is found in many epithelial tumors and is involved in tumor progression [50]. In vitro studies have shown that HIPK2 overexpression represses integrin alpha(6)beta(4) transcription that is conversely upregulated in colon cancer cells depleted of HIPK2 function by small interference RNA (siRNA) [48]. Immunohistochemistry analysis of HIPK2 nuclear and cytoplasmic compartmentalization performed in 67 stage I and II wtp53-carrying breast cancer patients showed that an high integrin alpha(6)beta(4) immunoreaction is correlated with HIPK2 cytoplasmic relocalization; in contrast, when tumors are integrin alpha(6)beta(4) negative, HIPK2 is localized in the nucleus [48]. These findings link HIPK2 activity to the suppression of the survival function induced by integrin alpha(6)beta(4) in agreement with other studies showing that depletion of HIPK2 function enhances in vivo tumorigenicity [51,52]. Regarding HIPK2 regulation by the proto-oncogenic non-receptor kinase Tyr kinase Src, it has been shown that it binds and phosphorylates HIPK2 at multiple Tyr residues. This interaction interferes with Siah-1-mediated HIPK2 degradation [42,43], redistributing HIPK2 from the nucleus to the cytoplasm. In this way, Src suppresses HIPK2-induced p53 phosphorylation and apoptotic activity [49]. Finally, in an in vitro study, the leukemogenic fusion protein CBF-β-SMMHC, which is associated with the development of acute myeloid leukemia (AML), was found to delocalize HIPK2 into filamentous structures in the cytoplasm, probably interfering with the tumor-suppressor function of HIPK2 [53].

Heat shock (HS) has been shown to modulate HIPK2 stability and nuclear/cytoplasmic localization: a sub-lethal heat shock induces HIPK2 cytoplasmic localization with impairment of p53 apoptotic activity, while a lethal heat shock induces HIPK2 nuclear localization with consequent p53 apoptotic activation [54], underscoring the key role of HIPK2 in stress-mediated cell death. HIPK2 is rarely mutated: the only HIPK2 mutations were reported by a study about human acute myeloid leukemias (AML) in which HIPK2 shows an aberrant nuclear distribution. Again, the impairment of HIPK2 activity negatively impacts on p53 apoptotic activity [55,56]. Loss of heterozygosity (LOH) at the HIPK2 locus 7q32.34 has been reported in human lung fibroblasts of patients affected by idiopathic pulmonary fibrosis (IPF) in which HIPK2 was found to be downregulated [57]. Another way to inhibit HIPK2 function is by reducing the binding with p53 and, therefore, its apoptotic activity, as happens, for instance, in human papillomavirus (HPV) infection [58]. HPV has been shown to play a causal role as a co-factor in the development of cutaneous squamous cell carcinoma (SCC) [59]. It was shown that upon DNA damage with ultraviolet radiation (UV), which is the main risk factor for SCC, HPV23 E6 interacts with HIPK2 and impairs its localization in the PML nuclear bodies (NB) [58] where HIPK2 associates with p53, phosphorylates it in Ser46, and induces its apoptotic function [20,29]. As a consequence, HPV23 E6 prevents HIPK2-induced p53 phosphorylation and apoptotic activity, suggesting that cutaneous HPV infected keratocytes may overcome UV-induced apoptosis and be causal in the early onset of skin carcinogenesis [58].

**Figure 2:**
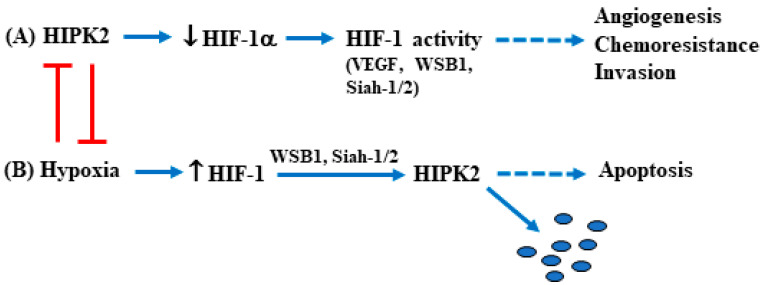
Schematic representation of the regulatory balance between HIF-1 and HIPK2 (red symbols) (see text). (**A**) HIPK2 activation inhibits HIF-1α expression (↓) and, therefore, HIF-1 activity, impairing the HIF-1-induced angiogenesis, chemoresistance, and tumor invasion (dotted blue arrow); (**B**) Hypoxia induces HIF-1 transcription (↑) which, through its targets WSB1 and/or Siah-1/2, induces HIPK2 proteasomal degradation (blue dots), impairing the HIPK2-induced apoptosis (dotted blue arrow).

A more common way to inhibit HIPK2 function is by inducing its proteasomal degradation, and, in this regard, a few ubiquitin ligases have been discovered. In response to mild DNA damage, it has been shown that p53-induced MDM2 can trigger HIPK2 protein degradation which, in turn, impairs p53 apoptotic activity. As a proof of principle, an HIPK2 mutant vector, resistant to MDM2 inhibition, is able to induce p53 apoptotic function [14]. Interestingly, in response to severe DNA damage, HIPK2 activation leads to MDM2 phosphorylation and protein degradation [60]. This autoregulatory loop between HIPK2 and MDM2 in response to mild/severe DNA damage consequently also affects p53 apoptotic/survival activity [31]. In normal conditions, HIPK2 is degraded by binding to the RING family ligase seven in absentia homolog-1 (Siah1). The DNA damage activates HIPK2 by releasing the binding with Siah1 which is phosphorylated by the damage-induced ATM/ATR [42,43]. TNF receptor-associated factor 2 (TRAF2) has also been shown to promote HIPK2 proteasomal degradation [61]. The promyelocytic leukemia (PML) gene, which encodes a putative tumor suppressor gene, interacts with multiple factors and their coactivators involved in the control of apoptosis [62]. Among them, it has been shown that PML activates transcription by protecting HIPK2 from SCF^Fbx3^-induced proteasomal degradation. In contrast, the leukemia-associated fusion protein PML-RARα induces the degradation of HIPK2 by inhibiting PML stabilization of HIPK2, underscoring a novel mechanism in the regulation of leukemogenesis [63]. Among other E3 ubiquitin ligases involved in HIPK2 degradation is WD40-repeat/SOCS box protein WSB-1 [64], a target of the hypoxia-inducible factor-1 (HIF-1) [65]; however, the ubiquitination and degradation of HIPK2 by WSB-1 is inhibited by genotoxic stress [64]. A hypoxic condition allows a markedly increased HIPK2 interaction with the RING family ligase Siah2, resulting in HIPK2 proteasomal degradation [66]. High-mobility group box 1 (HMGB1) is a conserved nuclear chromatin-binding protein that may have important tumor-promoting activity in several cancers, including hepatocellular carcinoma (HCC) [67]. It has been shown in vivo that HMGB1 promotes disease progression of human HCC [68]. At the molecular level, targeting HMGB1 inhibits Siah-2-induced HIPK2 ubiquitination and upregulates HIPK2 protein expression, suggesting that targeting HMGB1 could suppress HCC progression via HIPK2 activation [68]. Interestingly, HIPK2 overexpression binds to the HIF-1α promoter in a multiprotein co-repressor complex with histone deacetylase 1 (HDAC1), downmodulating both HIF-1α reporter activity and mRNA levels [51]. Hypoxia-inducible factor-1 (HIF-1) is a heterodimeric transcription factor that consists of two subunits, namely HIF-1α and HIF-1β. HIF-1β is constitutively expressed in cells, while HIF-1α stability is stimulated by hypoxia, growth factors, and several oncogenes [69]. Once activated, HIF-1 induces the transcription of several genes involved in many aspects of cancer progression, including angiogenesis, metabolic adaptation, apoptosis resistance, invasion, and metastasis [70]. HIPK2 overexpression reduces the basal levels of HIF-1α with a mechanism that does not involve hypoxia, inhibiting the HIF-1 transcriptional activity and, therefore, counteracting theHIF-1-induced angiogenesis and tumor progression, in vitro and in vivo [51,71,72,73]. In this way, the autoregulatory loop between HIF-1 and HIPK2 modifies the downstream pathways regulated by both proteins, and its balance may dictate the cancer survival/cell death outcome in response to therapies [71,72] (Figure 2).

Recently, it has been shown that microRNAs (miRNAs) may regulate HIPK2 both at the mRNA and the protein level, having an effect on cancer progression and angiogenesis as well as on other diseases [74]. For instance, circulating exomiR-1229 has been shown to target HIPK2 mRNA and downregulate it in colorectal cancer (CRC) tissues compared to the adjacent normal tissues [75]. A similar HIPK2 mRNA downregulation was previously found in CRC tissues compared to tissues of benign familial polyposis adenomas (FAP) [52]. ExomiR-1260b, derived from non-small cell lung cancer (NSCLC) has been shown to target HIPK2 in human umbilical endothelial cells (HUVECs) and promote the angiogenesis, migration, invasion, and chemoresistance of NSCLC cells [76]. The mechanisms of HIPK2 inhibition are summarized in Table 1.

### 2.1. HIPK2 Regulation by Hyperglycemia in Tumors

**Figure 3:**
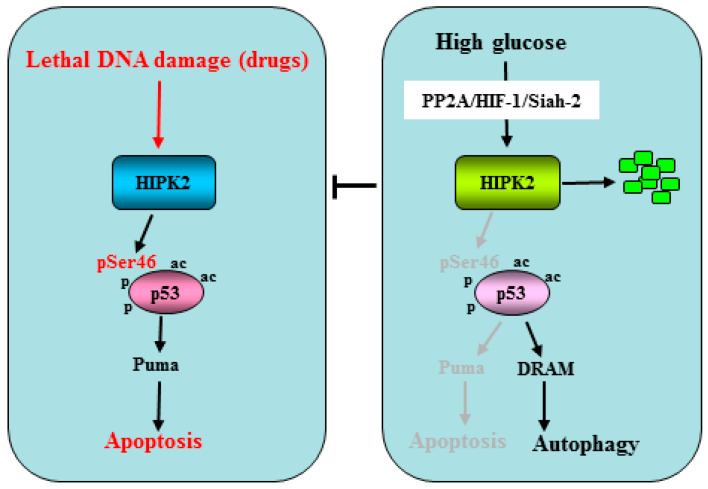
Schematic representation of HIPK2/p53 apoptotic regulation by HG. **Left panel**: lethal damage, such as that triggered by drugs, activates HIPK2 to induce p53 phosphorylation in serine 46 (pSer46) that consequently induces apoptosis through Puma transcription. **Right panel**: high glucose induces HIPK2 degradation (green squares) through the PP2A/HIF-1/Siah-2 axis, impairing the apoptotic activity of p53 that is instead switched toward transcription of the autophagic gene DRAM.

Taking advantage of the findings showing that hyperglycemia increases HIF-1α gene transcription [77] and induces the HIF-1 transcriptional activity irrespective of the oxygen levels [78], in an in vitro study, it was shown that high glucose (HG) condition promotes the degradation of HIPK2. Mechanistically, the HG-induced HIPK2 degradation was partly induced by the activity of the ubiquitin ligase Siah2, downstream of a protein cascade including protein phosphatase A2 (PP2A) and HIF-1α [79]. As a proof of principle, the HIPK2 degradation was confirmed by culturing the cancer cells in the presence of hyperglycemic sera derived from patients with T2D compared to normoglycemic sera [79]. HIPK2 protein stability regulated by the metabolic HG condition may impact on the cellular outcome: either survival or apoptosis. This can happen because the HG-induced HIPK2 inhibition consequently inhibits the p53Ser46 phosphorylation and the p53 apoptotic activity. Interestingly, the HG-mediated inhibition of the p53 apoptotic activity is counteracted by the phosphatase inhibitor of the PP2A named Calyculin A (CL-A) [80,81]. In addition, hyperglycemia increases autophagy in drug-treated cancer cells, in part depending on the p53 transcriptional switch from apoptotic target genes such as PUMA to autophagic genes such as DRAM [82] (Figure 3). As a proof of principle of the p53 transcriptional activity switch, xenograft tumors transplanted in normoglycemic mice displayed growth delay in response to chemotherapy, while those transplanted in diabetic mice did not, and such difference correlated with the PUMA to DRAM gene expression switch [82,83]. The different transcriptional activity of p53 could depend on the impairment of the HIPK2 kinase activity by the HG condition which, consequently, differentially affects the p53 transcriptional activity. Thus, the low/high genotoxic damage can differentially induce the p53 post-transcriptional modifications by modulating the upstream kinases, influencing the transcription of target genes: low damage induces mainly the cell cycle arrest-related or autophagic target genes, while high damage induces the apoptotic target genes [84,85]. The above findings highlight how metabolic conditions such as hyperglycemia might reduce the response to anticancer therapies by impairing the apoptotic activity of the HIPK2/p53 axis, in agreement with preclinical studies showing a reduction of chemotherapy cytotoxicity by hyperglycemia [86]. Interestingly, HG-induced HIPK2 degradation is counteracted in vitro by lowering the cell culture glucose amount, suggesting that this is a reversible effect [79]. Thus, an interesting strategy to pursue to keep the HIPK2/p53 axis functioning in cancer patients affected by diabetes or by other pathologies with hyperglycemia could be to reduce the glycemic load, although this hypothesis needs to be supported by further studies for clinical application.

The HG condition has been shown to trigger the antioxidant response, which correlates with the reduced Adriamycin-induced DNA damage and cell death, thus contributing to an increase in chemoresistance in cancer cells [87]. The master regulator of the antioxidant pathway is the transcription factor nuclear factor erythroid 2-related factor 2 (NRF2) [88,89] whose pharmacologic or genetic inhibition may re-establish cancer cell sensitivity to the chemotherapeutic drug cytotoxicity impaired by the HG [87,90]. The HIPK2/NRF2 relationship is quite intricate and still not completely unveiled. NRF2 may modulate HIPK2 both directly at the mRNA level and indirectly at the protein level. NRF2 has been shown to induce the HIPK2 mRNA transcription and engage with the HIPK2 protein, a pro-survival crosstalk to the detriment of the HIPK2 apoptotic activity [91]. Mechanistically, in this condition, HIPK2 has been shown to induce some antioxidant target genes which are in common with NRF2 (i.e., NQO1, HO-1) [91]. Moreover, NRF2 signaling has been shown to sustain the HIF-1 response by activating HIF-1α [92] and, consequently, although indirectly, to also modulate the HIPK2 function. Therefore, it is tempting to speculate that, in response to metabolic conditions that modify the redox state (e.g., hyperglycemia), NRF2 activation may calibrate the HIPK2 activation through, for instance, post-translational modifications [91] which impair HIPK2 apoptotic activity, favoring instead the transcription of genes promoting chemoresistance and tumor progression. This hypothesis, however, needs to be supported by further studies. HG may also promote tumor invasion [93] and epithelial–mesenchymal transition (EMT) in colon cancer cells in addition to promoting cancer cell migration and invasion [94]. Since HIPK2 has been shown to repress the vimentin gene promoter, one of the most important markers of EMT, and to restrain the cancer cell invasion [95], it is tempting to speculate that HIPK2 inhibition by the HG condition might also contribute to promoting tumor invasion.

Interestingly, it has been shown that cancer drug resistance, in part due to the HG-induced upregulation of hypoxic/glycolytic genes, such as HIF-1, GLUT-1, and HK-2, and to the inhibition of the JNK apoptotic pathway likely through NF-κB activation, can be counteracted by zinc(II) supplementation, which restores the drug cytotoxicity [77]. Zinc supplementation has been shown to play a role in cancer prevention [96], attracting studies for improving the effect of anticancer therapies. Thus, a genome-wide analysis in colon cancer cells has shown a reversal of the hypoxia-induced gene expression by zinc supplementation [97]. In addition, in vitro and in vivo murine studies have shown that zinc supplementation downregulates the HIF-1 activity and restores the cancer cell response to therapies through the re-establishment of the HIPK2/p53 apoptotic axis [71,72,98]. Zinc supplementation has been shown to ameliorate the severity of diabetic hyperglycemia and of the associated metabolic abnormalities, including hypoinsulinemia, insulin resistance, and the altered pancreatic morphology, in a murine model of diabetes [99]. Moreover, a systematic review and meta-analysis of randomized controlled trials reported that zinc supplementation significantly reduces several key glycemic indicators, particularly fasting glucose (FG), in subjects with diabetes [100]. Thus, zinc supplementation may offer a significant potential for clinical application in managing diabetic hyperglycemia and the related metabolic complications which promote cancer chemoresistance, in part by counteracting the HG-induced HIPK2/p53 inhibition.

### 2.2. HIPK2 and Diabetes Complications

**Figure 4:**
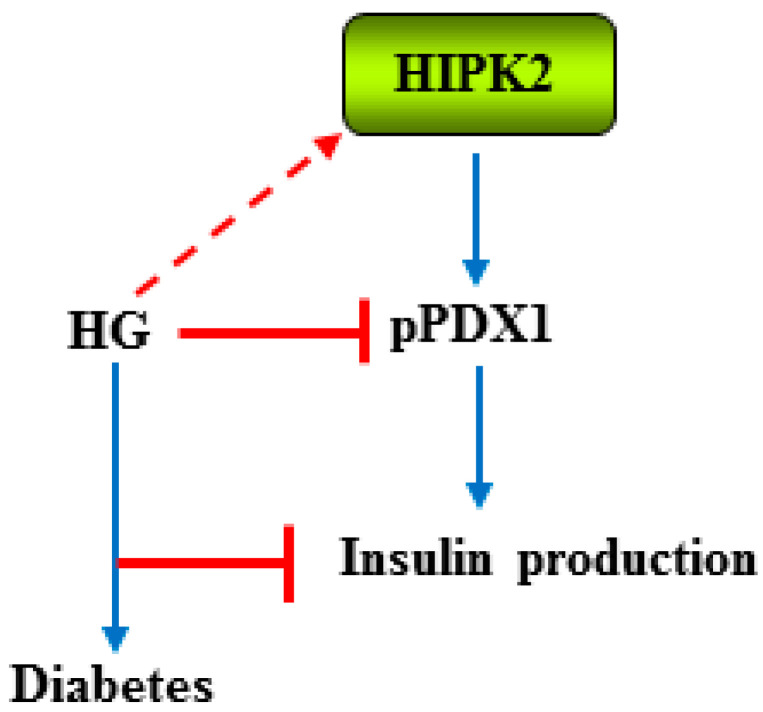
Schematical representation of the outcome of HIPK2-induced regulation of insulin promoter factor (IPF)-1/pancreatic duodenal homeobox (PDX)-1 transcription factor. HIPK2 phosphorylates and induces the transcriptional activity of IPF/PDX1 (blue arrow), which plays a crucial role in both pancreas development and maintenance of mature β-cell function for insulin production (blue arrow). High glucose (HG) inhibits PDX1 phosphorylation, likely through HG-induced HIPK2 inhibition (dot red arrow), which correlates with diabetes (blue arrow).

HIPK2 has been suggested to have a role in pancreatic development and mature β-cell function. Thus, it has been shown that HIPK2 is expressed in the pancreatic epithelium at an earlier stage of development, while it is expressed in the pancreatic endocrine cells at the later stage of development [101]. Mechanistically, the authors found that HIPK2 phosphorylates and induces the transcriptional activity of the insulin promoter factor (IPF)-1/pancreatic duodenal homeobox (PDX)-1, a homeodomain transcription factor which plays a crucial role in both pancreas development and maintenance of cell function, and that HIPK2 co-localizes with PDX1 in both the developing and adult pancreas [101]. A different study, using mass spectrometry and a phosphoserine-specific antibody, showed that HIPK2 regulates PDX1 function in living β-cells. Mechanistically, HIPK2 phosphorylates PDX1 at Ser-269, affecting the PDX1subnuclear distribution rather than the transcriptional activity [102]. Accordingly, a previous study has shown that HG reduces PDX phosphorylation in Ser-269 in primary rat islets [103]. The important role of IPF/PDX1 in diabetes has been shown in a mice model in which targeted disruption of the IPF1/PDX1 gene leads to overt diabetes with decreased insulin expression and secretion [104]. In humans, mutations in the IPF1 gene have been linked to diabetes [105]. The effect of high glucose in inhibiting PDX phosphorylation is also in agreement with a study showing that high glucose induces HIPK2 degradation [79]. The findings of HIPK2-mediated IPF1/PDX1 regulation highlight the key role of HIPK2 in glucose homeostasis in patients with diabetes [101] (Figure 4).

**Figure 5:**
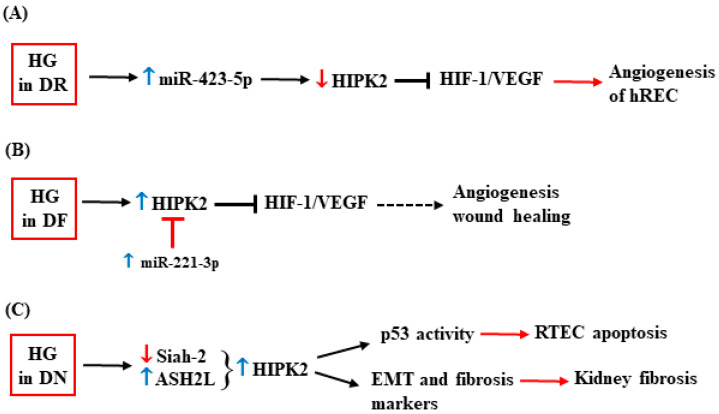
Schematic representation of HIPK2 regulation in diabetes complications. (**A**) High glucose (HG) induces overexpression (blue ↑) of miR-423-5p that targets and downregulates (red ↓) HIPK2: the HIPK2 downregulation de-represses the HIF-1/VEGF axis which induces angiogenesis in hREC (human retinal endothelial cells) (red arrow), contributing to diabetic retinopathy (DR). (**B**) HG induces overexpression (blue ↑) of HIPK2 in HUVECs (human umbilical vascular endothelial cells) which downregulates the HIF-1/VEGF-pathway, impairing angiogenesis and wound healing (dotted arrow) and contributing to diabetic foot ulcer (DFU). Upregulation (blue arrow) of miR-221-3p block HIPK2 (red symbol) counteracting the inhibition of angiogenesis induced by HG. (**C**) In diabetic nephropathy (DN), the overexpression (blue ↑) of HIPK2, as a consequence of Siah-2 inhibition (red ↓) or ASH2L upregulation (blue ↑), contributes to kidney fibrosis by p53-induced apoptosis of RTECs (renal tubular epithelial cells) (red arrow) and upregulation of epithelial–mesenchymal transition (EMT) and fibrosis markers (red arrow). See text for details.

One of the complications of diabetes is diabetic retinopathy (DR), which is also the primary cause of blindness in the world [106]. An interesting link between HIPK2 and DR has been depicted due to HIPK2 regulation by microRNA (miRNA), which is usually involved in DR vascular complications [107]. Taking advantage of a previous study in which the authors found that miR-423-5p levels are elevated in the plasma of DR patients [108], the authors, following this, evaluated the role of HG in this regulation. They found that the HG condition upregulates miR-423-5p, leading to the enhanced proliferation and angiogenesis of the retinal endothelial cells (hRCEs) as well as to the increased angiogenesis in the retina of a mouse model of streptozotocin (STZ)-induced diabetes [109]. miR-423-5p targets and downregulates HIPK2, with a consequent increase in the HIF-1α and VEGF protein levels, as corroborated by the knockdown of miR-423-5p that recovers the HIPK2 levels and suppresses the HG-induced angiogenesis [109]. The results indicate that the miR-423-5p-induced downregulation of HIPK2 contributes to the progression of DR through the activation of the HIF-1/VEGF angiogenic pathway (Figure 5A).

Hyperglycemia can cause neurological disorders and impaired angiogenesis, leading to reduced wound healing [110] and resulting in diabetic foot ulcers [111,112]. miR-221-3p has been found to alleviate diabetic ulcers [113] and to counteract the inhibition of angiogenesis by HG [96]. In that study, the authors found an increased HIPK2 expression in vitro in the HG-cultured HUVECs and in vivo in skin tissues of diabetic mice compared to the skin of non-diabetic mice [114]. Thus, HG-increased HIPK2 expression correlates with reduced angiogenesis and impaired wound healing, aggravating diabetic foot ulcers, a condition that can be ameliorated by treatment with miR-221-3p. Mechanistically, it was shown that HIPK2 is targeted and inhibited by miR-221-3p overexpression, which restores angiogenesis inhibited by the HG condition, therefore improving wound healing [114] (Figure 5B).

The HG-increased expression of HIPK2 was also observed in glomerular mesangial cells (GMCs) of a mouse model of diabetic nephropathy (DN) [41]. DN is a leading cause of end-stage renal disease (ESRD) and a threat to patients with diabetes [115]. HIPK2 has been found to play a role in DN and, in particular, in inducing kidney fibrosis [116]. In a mouse model of human immunodeficiency virus (HIV)-associated nephropathy (HIVAN), the authors found that HIV increases HIPK2 stability by inhibiting Siah-1-mediated HIPK2 proteasomal degradation. In that model, the HIPK2 activity leads to the apoptosis of the renal tubular epithelial cells (RTECs) by p53 apoptotic activation. In addition, HIPK2 activation leads to kidney fibrosis by activating, in kidney epithelial cells, the TGF-β–Smad3 and Wnt–Notch pathways and by inducing the expression of EMT markers, including αSMA (smooth muscle actin), collagen I, and fibronectin [116]. The authors confirmed the role of HIPK2 in inducing kidney fibrosis and also in focal segmental glomerulosclerosis (FSGS), DN, and IgA nephropathy (IgAN) [116] (Figure 5C). Therefore, targeting HIPK2 could be a valuable strategy to inhibit renal fibrosis during DN. As a proof of principle, it has been shown that the anti-helminthic drug phosphate niclosamide (P-NICLO) [117] can inhibit TGF-β-induced HIPK2 expression, resulting in the inhibition of the downstream pathways that are responsible for fibrosis, such as Smad, Notch, NF-kB, and Wnt/β-catenin [118].

The dysfunction of glomerular mesangial cells (GMCs) is a pathological feature of DN and the main contributing factor to glomerulosclerosis. In a previous study, the authors found that HG activates the transcription factor ASH2L, which enhances HIPK2 expression, contributing to fibrosis and inflammation in GMC [119] (Figure 5C). Thus, the inhibition of ASH2L suppresses HIPK2 expression in HG-treated mesangial cells, with a consequent decrease in fibrosis (e.g., FN, collagen 3, collagen 1, MMP9, α-SMA, and CTGF) and inflammation markers (e.g., IL-6 and IL-1β); in agreement, IL-6 and α-SMA were found to be downregulated by HIPK2 inhibition with small interfering RNA (siHipk2) [119]. Although the upregulation of HIPK2 in the kidneys of STZ-induced mice and leptin receptor-deficient db/db mice has been observed [119], further studies are needed to unveil how HIPK2 affects DN.

## 3. Conclusions

HIPK2 is a “bona fide” oncosuppressor molecule that exerts its anticancer activity mainly by triggering p53 apoptotic activity and by inhibiting hypoxia-induced angiogenesis. Recent developments have suggested that HIPK2 deregulation by hyperglycemia may play a role not only in the chemoresistance of various cancer cell types but also in the progression of some diabetes complications, likely involving the same HIPK2-triggered molecular pathways. In this review, we highlighted that increased antiapoptotic and pro-angiogenic activities are crucial mechanisms underlying chemoresistance mediated by HG-induced HIPK2 degradation largely mediated by HIF-1-induced mechanisms. In vitro research has revealed that lowering the glucose load or inhibiting the HIF-1 pathway may be a promising strategy to overcome the HG-induced chemoresistance by restoring the HIPK2/p53 axis, improving the efficacies of chemotherapeutic regimes. Both the suppression of HIF-1 activity by zinc(II) supplementation and the targeted knockdown of HIF-1α expression by siRNA significantly enhanced chemosensitivity in cancer cells and suppressed chemoresistant xenograft tumor growth in vivo. Advances have been made in understanding the mechanisms underlying chemoresistance in response to HG-induced HIPK2 inhibition, and further development of targeted therapies provides encouraging prospects for clinical application to optimize patient prognosis in different cancers. It is noteworthy that targeting HIF-1 activity may be a safer approach rather than re-activating HIPK2, whose apoptotic activity could have side effects in normal cells. Furthermore, whether therapy resistance dependent on HG-induced inhibition of HIPK2 activity is universal or specific for certain tumor types remains largely unexplored. Intriguingly, deregulated angiogenesis in human retinal endothelial cells (hREC), by means of activation of the HIF-1/VEGF pathway upon miR-423-5p-induced HIPK2 downregulation in diabetic patients, contributes to diabetic retinopathy. On the other hand, the upregulation of HIPK2 correlates with reduced angiogenesis and impaired wound healing, aggravating diabetic foot ulcers, a condition that can be ameliorated by treatment with miR-221-3p, which targets and inhibits HIPK2. The HIPK2 upregulation in in vitro and in vivo models of diabetic nephropathy unveiled a role of HIPK2 in kidney fibrosis by apoptosis of the renal tubular epithelial cells (RTECs) induced by p53 activation and by HIPK2-induced EMT markers. These findings, underscoring the involvement of HIPK2, provide new insights into the molecular mechanism of frequent diabetes complications. As a future perspective, the detailed molecular mechanisms of how hyperglycemia (de)regulates HIPK2 activity in diabetic complications warrant further investigation to unveil the cell type and context-dependent function/dysfunction of HIPK2 in this metabolic condition to reduce the clinical complications of the hyperglycemia.

## Figures and Tables

**Table 1:** Mechanisms of HIPK2 deregulation.

Molecules and Stimuli Involved	Mechanisms of HIPK2 Inactivation	Tissue	Cells	Ref.
Integrin alpha(6)beta(4)	Cytoplasmic localization	+	+	[48]
Src	Cytoplasmic localization		+	[49]
CBF-β-SMMHC	Cytoplasmic localization		+	[53]
HPV23 E6	Impairment of PML-NB localization	+	+	[58]
Sub-lethal heat shock	Cytoplasmic localization		+	[54]
Gene mutation (in AML)	Downregulation	+	+	[55,56]
LOH (in IPF)	Downregulation	+		[57]
Overexpressed ExomiR-1229	Downregulation	+	+	[75]
Overexpressed ExomiR-1260b	Downregulation	+	+	[76]
MDM2	Protein degradation		+	[14]
Siah-1	Protein degradation		+	[42,43]
Siah-2	Protein degradation		+	[66,68]
PML-RARα	Protein degradation			[63]
TRAF-2	Protein degradation		+	[61]
WSB-1	Protein degradation		+	[64]

+: experiments performed in cell lines (Cells) and/or in tissue samples (Tissues).
